# Factors associated with antenatal mental disorder in West Africa: A cross-sectional survey

**DOI:** 10.1186/1471-2393-11-90

**Published:** 2011-11-04

**Authors:** Bola Ola, Jim Crabb, Adetokunbo Tayo, Selena H Gleadow Ware, Arup Dhar, Rajeev Krishnadas

**Affiliations:** 1 Department of Behavioural Medicine, LASUCOM Ikeja, Lagos, Nigeria; 2 Forth Valley Royal Hospital, Stirling Road, Larbert, UK; 3 Department of Obstetrics and Gynaecology LASUCOM Ikeja, Lagos, Nigeria; 4 Riverside Resource Centre Community Mental Health Team, 8 Sandy Rd, Glasgow, UK; 5 Sackler Institute of Psychobiological Research, Section of Psychological Medicine, Southern General Hospital, Glasgow, UK

## Abstract

**Background:**

Maternal mental illness is likely to have a profound impact in less developed parts of the world. A mother experiencing mental illness in a low income setting is at risk of providing sub-optimal care for her offspring which can have grave consequences in an environment where poverty, overcrowding, poor sanitation, malnutrition, tropical diseases and a lack of appropriate medical services may be pronounced. Given the profound consequences of antenatal and postnatal mental illness on maternal mental health, foetal wellbeing and childhood growth and development the factors associated with mental illness in a Sub-Saharan setting merit clarification and investigation.

**Methods:**

A prospective survey design was conducted in Lagos. Self reporting questionnaire 20 items - SRQ20 - assessed the presence of mental illness. The WHO Multi-country Study on Women's Health and Domestic Violence Questions assessed women's exposure to violence. Numerous variables potentially associated with mental illness including maternal socio-economic factors, maternal characteristics, obstetric variables and the characteristics of previous children were recorded. Direct logistic regression was performed to assess the impact of a number of variables on the likelihood of presence of mental disorder in the population.

**Results:**

189 women were surveyed. 7% met the criteria for experiencing a common mental disorder according to their score on the SRQ-20. Of variables examined only the number of female children and the presence of inter personal violence predicted being a case of mental illness (OR = 3.400; 95%CI = 1.374 - 8.414 and OR = 5.676; 95%CI = 1.251 - 25.757 respectively).

**Conclusions:**

Rates of mental disorder found in our study were lower than those previously observed internationally and in Africa, perhaps reflecting stigma about disclosing symptoms. The predictive nature of violence on mental disorder is in keeping with international evidence. Our study demonstrated that exposure to inter personal violence within the last 12 months and increasing numbers of female children predict the presence of mental illness in a sample of pregnant Nigerian women. Training and education for primary health care and obstetric health workers should highlight these areas.

## Background

There is increasing evidence that anxiety and depression are more prevalent in late pregnancy compared to the postpartum period and an increasing body of evidence highlights the role of antenatal stress on maternal mental health and childhood growth and development [1]. Pre-natal stress has been identified as referring to a range of events from exposure to traumatic events, self-reports of daily hassles and negative life events during pregnancy [2]. A number of independent studies have found associations between antenatal stress and adverse neurobehavioral outcomes including impaired social, emotional and cognitive functioning during childhood [3]. Antenatal stress has also been associated with preterm delivery and low birth weight, poor attendance at antenatal clinics as well as elevated risk of cardiovascular disease later in life [4-6].

Maternal stress and mental illness is likely to have a profound impact in less developed parts of the world. A mother experiencing mental illness in a low income setting is at risk of providing sub-optimal care for her offspring which can have grave consequences in an environment where poverty, overcrowding, poor sanitation, malnutrition, tropical diseases and a lack of appropriate medical services may be pronounced [7,8]. Adewuya et al in their report on the impact of postnatal depression on infants' growth in Nigeria, found statistically significant poorer growth rates in infants of depressed mothers, compared to non-depressed mothers at 3 and 6 months postpartum [9]. Depressed mothers were found to be more likely to stop breastfeeding earlier and their infants were more likely to have episodes of diarrhoea and other infectious illnesses.

Given the profound consequences of antenatal and postnatal mental illness on maternal mental health, foetal wellbeing and childhood growth and development in Sub Saharan Africa it is important as a first step to ascertain the scale of the problem, particularly as epidemiological studies have been scarce in the region. Once this has been established it will also be vital for policy makers and clinicians to be able to identify those at risk of developing mental disorder in pregnancy so that preventative measures can be implemented.

## Methods

### Sampling

Our study had a prospective survey design and was conducted in Lagos, the largest city in Nigeria. Lagos has an estimated population of 15 million and has two teaching hospitals with psychiatric facilities and one psychiatric hospital. Consecutive pregnant women attending the antenatal clinic at the maternity clinic of the Lagos State University Teaching Hospital, Ikeja, for routine appointments over an eight week period were interviewed. Non pregnant women, women with other health problems attending clinic and family members were excluded. We estimated sample size using a method proposed by Daniel [10]. International systematic reviews have shown the prevalence of antenatal depression to be 7.4-12% [11]. Assuming that the prevalence rate of mental disorder in Nigeria would be 10%, using a 95% confidence interval and a precision of 5% we calculated a sample size of 144.

### Procedures

Participants gave written informed consent which was based on the procedure used by the WHO Multi-country Study on Women's Health and Domestic Violence [12]. Consent forms were approved by local health workers and service users for acceptability. Ethical approval was granted by the Ethics and Research Committee of Lagos State University Teaching Hospital, Ikeja. All subjects were able to read, write and converse fluently in English and completed the following measures.

### Measures

In order to measure the presence of mental illness our study employed the self reporting questionnaire 20 items (SRQ20). This consists of twenty yes/no questions with a reference period of the previous thirty days. It has acceptable levels of reliability and validity in developing countries and is recommended by the World Health Organisation (WHO) as a screening tool [13]. It has previously been used to measure maternal illness in a four country study which included Ethiopia where a cut off score of 7/8 was used to separate probable non-cases/cases of common mental disorder [14].

The WHO Multi-country Study on Women's Health and Domestic Violence Questions were used to assess women's exposure to violence [12]. Rather than being an exhaustive list a limited number of questions about specific acts that occur in violent partnerships are covered. This approach has been found to encourage greater disclosure of violence. Psychometric analysis has been performed on the terms used in the questionnaire which were found to be valid and reliable measures for each type of violence. The WHO ethical and safety guidelines for domestic violence research were adhered to throughout our study [15]. Practices developed in the WHO Multi-country Study on Women's Health and Domestic Violence to ensure confidentiality, minimize distress and provide support to women experiencing inter personal violence were also implemented [12].

Our literature review highlighted numerous variables potentially associated with mental illness including maternal socio-economic factors, maternal characteristics, obstetric variables and the characteristics of previous children [12,14]. Details regarding these variables were recorded in our sample population. Raters were trained to use manualised versions of the above rating scales and questionnaire. Two raters assessed the same thirty cases and the inter-rater reliability was found to be acceptable with a kappa's coefficient of 0.89.

### Statistical methods

Statistical analysis was done using SPSS version 17 [16]. Binary logistic regression was performed to assess the impact of a number of variables on the likelihood of presence of common mental disorder caseness in the population. The dependent variable was caseness based on a cut off score of 7/8 on the SRQ 20 scores. We tested the effects of 9 independent variables, based on previous studies. They were age, household income, receiving help from partner, number of previous female children, history of physical violence in last 12 months, perceived difficulty meeting daily needs, primi-parity, planned current pregnancy and a history of substance use.

## Results

200 women attended the clinic over the 8 week period. 189 were surveyed, 11 refused to participate giving a response rate: 94.5%. Demographic and obstetric details are shown in Table 1.

**Table 1:** Demographic and obstetric factors

Age [mean; s.d.]		30.28 (4.4)
Primipara [n;%]		52 (27.5%)

Number of previous female kids [mean; s.d.]		0.45 (0.66)

Gestational age [mean; s.d.]		26.59 (4.65)

History of IPV in the past 12 months [n;%]		18 (9.5%)

Difficulty meeting needs [n;%]		41 (21.7%)

Unplanned pregnancy [n;%]		29 (15.3%)

History of substance use [n;%]		21 (11.1%)

Patient's income [n;%]	Less than 20000	49 (25.9%)

	20000-49999	97 (51.3%)

	50000-79999	40 (21.2%)

	80000 - 99999	2 (1.1%)

	100000 -above	1 (0.5%)

Partner's income [n;%]	Less than 20000	3 (1.6%)

	20000-49999	50 (26.5%)

	50000-79999	94 (49.7%)

	80000 - 99999	31 (16.4%)

	100000 -above	11 (5.8%)

No perceived help from partner [n;%]		37 (19.6%)

History of Higher Education [n;%]		142 (75.1%)

13 women interviewed (7%) met the criteria for experiencing a common mental disorder according to their score on the SRQ-20. With regards to physical violence, 12.2% (23) of women had ever been exposed to violence. 9.5% (18) had experienced physical violence within the last 12 months whilst 5.3% (10) were currently suffering this form of abuse. Our analysis focused on interpersonal violence exposure within the last 12 months as this period would correspond with all of the current pregnancy.

There were no differences between cases and non-cases on age (MW = 1035.0; p = 0.56) and gestational age of current pregnancy (MW = 1000.5; p = 0.45). All patients were either in their second or third trimesters (range = 16 - 36 months). Results of the binary logistic regression predicting caseness is shown in table 2. The model containing all 9 predictors was statistically significant (χ^2 ^= 16.9; df = 9; p = 0.05), indicating that the model was able to distinguish between cases and non-cases. The model as a whole explained around 22% of the variance in caseness. As shown in table 1, the strongest predictor was a history of physical violence in the last 12 months, recording an odds ratio of 5.67. This indicated that those who experienced physical abuse during the last 12 months were over 5 times more likely to be a case than those who were not physically abused, controlling for all other factors in the model. The odds ratio of 3.4 for the number of previous female children indicated that for every additional previous female child in the family, a woman was 3 times more likely to have a common mental disorder. None of the other variables predicted caseness. After adding gestational age into the model, it failed to be statistically significant (χ^2 ^= 16.94; df = 10; p = 0.076; Nagelkerke R^2 ^= 0.218).

**Table 2:** Logistic regression predicting caseness on SRQ20

	B	S.E.	Wald χ^2^	Sig.	Odds Ratio (95% C.I)
Age	-.045	.078	.328	.56	0.956 (0.821 - 1.114)

Household income	-.074	.171	.187	.66	0.929 (0.665 - 1.298)
Receiving no help from partner	1.084	.805	1.815	.17	2.956 (0.611 - 14.307)
Number of previous female children	1.224	.462	7.007	.008	3.400 (1.374 - 8.414)

History of physical violence in last 12 months	1.736	.772	5.062	.024	5.676 (1.251 - 25.757)

Perceived difficulty meeting daily needs	.471	.691	.465	.49	1.602 (0.413 - 6.206)
Primi-para	1.162	.865	1.805	.18	3.196 (0.587 - 17.406)

Planned current pregnancy	.498	.806	.381	.54	1.645 (0.339 - 7.986)

History of substance use	-.074	.966	.006	.94	0.929 (0.140 - 6.173)
Constant	-2.942	2.376	1.534	.216	.053

Dependent variable: Caseness (1); Model χ^2 ^= 16.9; df = 9; p = 0.05; Nagelkerke

R^2 ^= 0.22. All categorical risk factors are coded as "0" for absence of risk factor and "1" for presence of risk factor.

## Discussion

Systematic reviews of studies in Europe and North America have shown the prevalence of antenatal depression at the first, second, and third trimesters to be 7.4, 12.8, and 12%, respectively with major depression affecting up to 12.7% of women [11,17]. A recent comprehensive review of the literature regarding pre and post natal mental health in Africa reported rates of antenatal depression and anxiety of 11.3-13.1% and 14.8% respectively across the continent [18]. Our study found lower rates of mental disorder of 7%, a figure comparable with other studies examining maternal mental health in Nigeria, which identified depression rates of 8.3-10.3% [19,20]. The disparity between rates of mental illness in Nigeria and Africa as a whole may in part be explained by the particularly high rates of stigma regarding mental illness in West Africa, which might prevent a woman disclosing symptoms of mental illness. This interpretation is supported by our findings that none of the women in our study reported a previous personal or familial history of mental illness. Typical West African cultural explanations for mental disorder include criminality, spirit possession & punishment accompanied with fears that individuals with mental illness are mentally retarded and/or dangerous [21]. In the context of pregnancy these misconceptions may be particularly stigmatising as women may fear that disclosing a past or familial history of mental illness could suggest that their child may be already damaged by association with mental illness. With regards to anxiety symptoms our results were lower than another study by Adewuya et al which found rates of anxiety of 39% in pregnant Nigerian women compared to 16.3% in the non pregnant population [19]. This difference may reflect the differences in methodology, particularly the rating scale used. In the work by Adewuya et al anxiety was measured according to DSM-IV criteria whilst the SRQ-20 employed in our study measures a combination of mood and anxiety symptoms (see limitations).

Recent systematic reviews of the literature from the United States, Canada, Europe, Australia and New Zealand have identified the following risk factors for depressive symptoms in pregnancy; maternal anxiety, life stress, history of depression, lack of social support, unintended pregnancy, Medicaid insurance, domestic violence, lower income, lower education [22]. The following factors were found to have no association with antenatal mental illness; smoking, alcohol use, illicit drug use, parity, maternal race/ethnicity, maternal age and obstetric history (including spontaneous abortions, elective abortions, and foetal deaths in-utero) [22]. Adewuya's studies in West Africa identified correlates of antenatal anxiety including younger age, primiparity and the presence of medical problems as well as risk factors for antenatal depression including hospital admissions during pregnancy; female sex of the baby; preterm delivery; instrumental delivery; caesarean section, previous still birth, perceived lack of social support and being single/separated or polygamous. Esimai's later study which measured fewer variables found that a low level of partner intimacy and a lack of financial support were associated with antenatal depression and anxiety [20]. Aside from these studies there is inconclusive evidence from studies across Africa that socioeconomic variables are implicated in antenatal mental health [18]. Our study only found an association between mental disorder and one of these variables, the number of previous female children. The sex of the child has been hypothesized as a potentially important risk factor in certain less developed parts of the world where there may be social and cultural pressures on women to give birth to sons [23]. This is particularly the case in Nigeria where women are held culpable for the sex of the baby and lack of a male heir is often cited as a reason for relationship break down [24].

To date we are not aware of any other work which has examined the relationship between antenatal mental illness and exposure to interpersonal violence in West Africa. Our finding of a positive relationship between violence and symptoms of mental disorder is in keeping with previous evidence. Bivariate studies have shown a small association between domestic violence and depressive symptoms whilst multivariate studies show a small to medium association [22]. In 1 study of 128 women in North America, a history of abuse within the past year was associated with almost 2.5 times the odds of a positive screen for depression [25]. Similarly work from North America and Europe has established a strong association between suicidality and IPV exposure [26,27]. Though our results had a wide confidence interval this most likely reflects the small prevalence of caseness

### Limitations

The use of an arbitrary cut off point on the SRQ-20 to define a 'case' of mental disorder may have introduced measurement bias and could explain the sharp difference between cases of anxiety found in Adewuya's study and cases of mental illness found in our own [19,28]. Inadequacies in the ability of the SRQ 20 to detect mental disorder in a Nigerian population may explain our low rate of caseness. The scale has however been validated in a number of Sub Saharan African countries. In the Uganda a cut of score of 6 has been used to denote psychological distress [29]. A more recent validation study in Rwanda, published after our data had been recorded suggested the scale had different cut off points for men and women, with ten being identified in females [30]. In the absence of a validation study from West Africa our use of 8 as a cut off point seems reasonable based on the evidence available.

A number of challenges exist in implementing antenatal care in Africa. Late booking is particularly common in Nigeria, particularly in multiparous women, and has been found to be associated with educational level of partners, previous miscarriage and medical problems in the index pregnancy [31]. It has been suggested that most Nigerian woman wish to give birth on their own because of the pride associated with unassisted delivery and the loss of esteem associated with operative interventions [32]. It is possible then that selection bias was present in our study as our sample consisted of woman motivated to attend medical clinics in an urban area. All women in our study could also read, write and speak English therefore our results may not be generalisable to rural populations where this is not the case. Our sample does perhaps represent the evolving face of West Africa that is experiencing increasing urbanisation and socio-economic development. This may have influenced our findings in several ways. It is possible that an urban, educated population might have more progressive views about female offspring, mental illness and the role of obstetric care, compared to their rural counterparts. Pregnant women in rural facilities outside of the mainstream health system may be therefore less likely to report levels of mental disorder due to greater levels of stigma. An alternative explanation is that women in urban areas are more vulnerable to psycho-social stresses similar to those that women in higher income countries experience relating to social change, including urbanisation and the breakdown of family networks and traditional support systems. If this were the case we would expect to find differences in levels of mental disorder in urban compared to rural samples. Such data is however lacking from the existing evidence base. Firm conclusions about the potential bias caused by our sample are therefore impossible at this stage. Our study contributes to the emerging view that there are characteristics of maternal mental illness unique to Sub Saharan Africa. Further research is however required to compare rates of common mental disorder and risk factors in pregnant women from different geographical areas and socioeconomic backgrounds within the region.

## Conclusions

Our study demonstrated that exposure to physical violence within the last 12 months and increasing numbers of female children predict the presence of mental illness in a sample of pregnant Nigerian women. We suggest that questions regarding mental illness and inter personal violence should be included in standard obstetric screening in Sub Saharan Africa. More detailed psychiatric screening including thoughts of suicide should be considered for any woman reporting exposure to inter personal violence. Women with multiple female offspring should also be screened for mental illness in Sub Saharan Africa. Training and education for primary health care and obstetric health workers should highlight these areas. Although no causative links can be inferred from the results of our study, broader community and population approaches to addressing inter personal violence and cultural expectations of infant gender may be required, to reduce the psychosocial and cultural stressors pregnant women face, that may increase vulnerability to mental illness during the perinatal period. It is likely that successful interventions to improve maternal mental health in Sub Saharan Africa need to involve political, social, and educational approaches, as well as ensuring appropriate screening by healthcare professionals.

## Abbreviations

SRQ: Self reporting questionnaire 20 items; WHO: World Health Organisation; O.R: Odds ratio;

## Competing interests

The authors declare that they have no competing interests.

## Authors' contributions

BO and AT were involved in the design and implementation of the study including data collection. JC devised the study design and wrote the paper. SG & AD conducted the literature review. RK performed the statistical analysis and helped preparing the manuscript. All authors contributed to and approved the final draft of the text.

## Pre-publication history

The pre-publication history for this paper can be accessed here:

http://www.biomedcentral.com/1471-2393/11/90/prepub
